# In their own words: qualitative study of high-utilising primary care patients with medically unexplained symptoms

**DOI:** 10.1186/1471-2296-10-67

**Published:** 2009-09-21

**Authors:** Francesca C Dwamena, Judith S Lyles, Richard M Frankel, Robert C Smith

**Affiliations:** 1 Department of Medicine, Michigan State University, East Lansing, MI, USA; 2 Michigan Department of Community Health, Lansing, MI, USA; 3 Health Services Research and Development Center of Excellence, Roudebush VAMC, Department of Medicine, Indiana University, Indianapolis, IN, USA

## Abstract

**Background:**

High utilising primary care patients with medically unexplained symptoms (MUS) often frustrate their primary care providers. Studies that elucidate the attitudes of these patients may help to increase understanding and improve confidence of clinicians who care for them. The objective of this study was to describe and analyze perceptions and lived experiences of high utilising primary care patients with MUS.

**Methods:**

A purposive sample of 19 high utilising primary care patients for whom at least 50% (69.6% in this sample) of visits for two years could not be explained medically, were encouraged to talk spontaneously about themselves and answer semi-structured questions. Verbatim transcripts of interviews were analyzed using an iterative consensus building process.

**Results:**

Patients with MUS almost universally described current and/or past family dysfunction and were subjected to excessive testing and ineffective empirical treatments. Three distinct groups emerged from the data. 1) Some patients, who had achieved a significant degree of psychological insight and had success in life, primarily sought explanations for their symptoms. 2) Patients who had less psychological insight were more disabled by their symptoms and felt strongly entitled to be excused from normal social obligations. Typically, these patients primarily sought symptom relief, legitimization, and support. 3) Patients who expressed worry about missed diagnoses demanded excessive care and complained when their demands were resisted.

**Conclusion:**

High utilising primary care patients are a heterogeneous group with similar experiences and different perceptions, behaviours and needs. Recognizing these differences may be critical to effective treatment and reduction in utilisation.

## Background

Medically unexplained symptoms (MUS) are physical symptoms with little or no underlying organic disease [1]. Many patients with MUS consume healthcare disproportionately as they seek help to ease their suffering. Perceived or actual experiences of scepticism and distrust in medical consultations [2-4] and with family members and co-workers [3] complicate their lives. Some patients use strategies like somatisation to engage with the healthcare system, and mystifying and martyrising to manage their health [5]. Unfortunately, the resulting high utilisation can be very expensive [6] and rarely is productive. Patients with persistent MUS report more psychological distress, functional impairment, and social isolation than non-MUS patients with similar utilisation [7].

Primary care providers, who are the preferred consultants [8] for these unfortunate patients are equally frustrated [9,10], and many lack confidence in their ability to provide adequate care for these difficult patients [11]. Even practitioners with more optimistic attitudes about their skills report significant barriers to implementing one method of treating MUS in primary care [12]. Mutual frustration between physicians and patients is fuelled further by differing goals. Patients typically seek emotional support [13,14] often by providing psychosocial cues during consultations [14]. Doctors, who focus primarily on symptom alleviation [13] often ignore these cues [14] and unwittingly promote further somatisation [15]. Moreover, some patients with MUS avoid discussing important psychosocial concerns with providers to avoid diverting them from thoroughly considering organic causes [16].

Much of the frustration surrounding treatment of MUS relates to difficulties with definition and diagnosis. Medically unexplained symptoms have traditionally been classified with the Diagnostic and Statistical Manual (DSM) in Psychiatry or as one of the functional somatic syndromes in medical specialties [17]. Although helpful for research purposes, these classifications have been too restrictive to be useful to most primary care providers. Rather than distinct MUS entities, primary care providers encounter nebulous physical and psychological ailments on a much wider continuum of severity, duration and co-morbidity [12,18]. To identify more clinically relevant samples, previous studies have relied on primary care providers [16,19] or consultants [20] to identify patients with MUS. This strategy may exclude important subsets of MUS patients such as those with self-limiting symptoms or those with significant medical or psychiatric co-morbidity [21,22]. Moreover, the perceptions and experiences of patients with unlabelled MUS may be quite different from the contentious experiences [3,23] of labelled patients [20].

An innovative chart review method [24] allowed us to identify typical high utilising primary care patients for whom MUS accounted for at least 50% of visits for two consecutive years [24]. Using a priori definitions, trained physicians rated symptoms for each visit as "documented organic," "documented non-organic" if sufficient diagnostic testing was negative, or as "undocumented" if there was either insufficient, or no diagnostic testing. Visits were rated as MUS if 50% or more of all symptoms for that visit were either documented non-organic or undocumented. Patients were considered to have severe MUS if at least 25% of MUS visits were documented non-organic and moderate MUS if less than 25% of MUS visits were documented non-organic.

We conducted the qualitative study reported here to provide a contextual description of this unique sample of patients with MUS where more than 75% did not qualify for a DSM derived diagnosis [22]. We sought to better understand the attitudes and lived experiences of these distressed, high utilising primary care patients with MUS, who may or may not have been labelled with a specific MUS diagnosis.

## Methods

To achieve our goal we used qualitative, Grounded Theory methods [25-27] to generate, elaborate, and refine emerging categories from verbatim transcripts of in-depth interviews with 19 MUS high utilising MUS patients. The Institutional Review Board at Michigan State University approved the study, and all patients gave informed consent.

### Participants - Sampling Strategy

Like Travers et al [3], we adopted several sampling procedures to obtain data that reflect the scope and patterns of typical high utilising patients with MUS in primary care. Elsewhere, we have described how we identified patients for a randomized controlled trial (RCT) of treatment for primary care MUS patients in a large health maintenance organization in Michigan in 2000 [28,29]. Control patients from this trial provided an initial pool of potential participants with varied combinations of chronic, persistent unexplained symptoms and minor self-limiting complaints that did not individually require extensive testing. From this pool, JSL and RS used maximum variation sampling to select participants who reflected key variables: chart-rated MUS severity, age, and gender. Concurrent collection and analysis of data enabled us to use theoretical saturation to provide a final sample of 19 patients out of the 23 patients approached. The final sample included three males and 16 females. Nine were married, and fourteen had at least two years of college. Mean age was 48 years with a range of 31 years to 65 years. Nine had severe and 10 had moderate chart-rated MUS. Seven were previously labelled with MUS syndromes (5 fibromyalgia, 2 IBS) and twelve had not previously been labelled. Compared to other control patients, included patients had a higher percentage of MUS visits (69.6% vs. 60.4%, p = 0.042). There was no difference in age, gender, mean number of visits/year, or proportions of moderate and severe chart-rated MUS. Interviews were conducted from November 2001 to October 2002.

### Data collection and analysis

The interviewer (FCD) was trained in qualitative interviewing and had no prior relationship with any participant. She explained to participants that the goal of the study was to understand the experiences and perceptions of patients with multiple clinic visits. Using a semi-structured questionnaire (Table 1), she began each interview with an open-ended inquiry that allowed participants to determine the content, pace, and sequencing of the interview for 30 - 45 minutes. If the following topics had not arisen, she asked participants about their explanatory models [30]; locus of control [31]; health-seeking behaviour [32]; abuse; gender effects; relationships; and expectations for the future. These topics were identified a priori and over the course of the study from newly evolving themes we identified. As FCD reviewed the transcripts of each interview, she made changes in subsequent interviews based on emerging themes. For example, after the first two participants talked spontaneously about childhood distress and abuse, she asked about those topics in the remaining interviews. Similarly, after analyzing the first five interviews, she asked follow-up questions whenever patients spontaneously brought up the topic of religion. She also sought subsequent interviewees' (male and female) opinions about the influence of gender on doctor-patient relationships when the narrative of one of the participants (a man) suggested it might be important. This style of "progressive" interviewing is characteristic of qualitative discovery-oriented research. All interviews were audiotaped and were transcribed verbatim. The transcripts, stripped of patient identifiers, were used to conduct further analysis.

**Table 1:** Semi-structured questionnaire for interviews

**Category**	**Instructions**
Introduction	• Introduce self
	• Introduce the study
	• Introduce the interview (audiotape, notes)
	• Pause for questions
Open-ended	• Say: "Tell me about yourself"
beginning (30-45 minutes)	• Use patient-centered method (patient-directed, empathic) to expand the patient's story of the physical, personal and emotional aspects of their illness
Directive Questioning (30-45 minutes) Ask these questions if the corresponding topics have not been discussed, continue to expand newly raised topics with clarifying questions and patient:	
Explanatory	• "What do you believe is the root cause of your problems?"
models	• "What are your concerns about your problems?
	• How have your health problems affected your life?"
Locus of control	• "Who do you feel has the most control over your health (life)?"
	
Health-seeking behaviour	• "Whom do you turn to for information about your health? Is there anyone else?"
	
Relationships	• "How is your relationship with your healthcare provider?"
	• "What are your expectations when you go to the doctor?"
	• "Do your issues get resolved to your satisfaction?"
	• "How satisfied are you with the care you have received?"
	• "How does gender affect your relationship with doctors?"
Expectations for the future	• "What are your expectations about your future?"
	
**Added after Interview 2** Ask this question if not already discussed	
Describe your childhood	• "Have you ever been abused?"

We used an iterative consensus-building process [25-27] similar to the immersion/crystallization method described by Crabtree and Miller [33] to ensure that further analysis was grounded in the data rather than based on our own pre-existing groupings or framework. The multidisciplinary team comprised researchers and clinicians from primary care, psychiatry, sociology, and communication. This use of authors from different disciplinary backgrounds is an established procedure for improving validity in qualitative studies. FCD, JSL, and RCS identified *preliminary themes *(see Table 2) by independently reading, taking notes, and verifying concepts from the first five transcripts. We reconciled differences, clarified, and refined categories by consensus and then developed *working themes *(see Table 2) by testing preliminary themes against a second set of five transcripts. With the working themes in hand we read and discussed the remaining nine transcripts to further identify, refine, and elaborate previous themes and to identify any new themes that emerged. Finally, we reread all 19 transcripts, developing and clarifying relationships in categories; and independently verifying our final themes.

**Table 2:** Preliminary and working themes

**Preliminary themes**	**Working themes**
*1. Behaviour/Action* Pleasure in life, Coping/Dysfunction, Job satisfaction *2. Primary relationships* Duration of marriage *3. Secondary relationships* *4. Doctor-patient relationship* *5. Mechanism of illness* Identity/invisible, Stage of development, Personality, Locus of control, Number of siblings, Location of patient, Abuse, Family History, Explanatory model *6. Physical Symptoms* Fear of physical disease, Care seeking, Secondary gain *7. Diagnosis* Medical (primary or secondary), Psychiatric diagnosis (primary or secondary), MUS diagnosis (minor acute, somatisation, neither) *8. Emotionality* Expression during interview, Evidence of emotionality in life, Insight/psychological savvy *9. Excessive testing/medicalisation* *10. Reaction to interview and study* *11. Religion/spirituality* *12. Healthcare system* *13. Litigation* *14. Education/training*	*1. Primary mechanism* *2. Secondary gain* *3. Insight (mind/body connection)* *4. Emotionality* *5. Symptoms focus* *6. Fear of physical disease* *7. Quality of dominant relationships* *8. Obesity*

## Results

We identified eleven final themes and three patient groups. The eleven final themes fell into three broad categories (see Table 3) which we defined: a) "experiences" as participants' actual descriptions of events that occurred in their lives; b) "perceptions" as attitudes and/or insights; c) "behaviours" as actions of participants that were observed during the interview, or were inferred from their narratives. Three patient groups with discrete patterns of consultation emerged also, when we re-read transcripts to clarify concepts and test emerging theories. We used the same iterative and consensus-building process described above to test and to assign group membership, and to settle on the following names for the three patient groups: a) coping high utilisers; b) classic high utilisers; and c) worried high utilisers. We then described the three groups using the 11 themes and supplemented group descriptions with previously collected demographic and clinical data. Finally, we reread all transcripts to discern reasons for MUS and high utilization in each of the patient groups.

**Table 3:** Final themes (n = 11) with definitions and participants who were coded as demonstrating the theme

**Theme**	**Participant ID**
**Experiences**	
*1. Impact of childhood trauma* Expressions of traumatic experiences at a young age	3, 4, 6, 7, 8, 9, 13, 14, 15, 19
*2. Impact of adult abuse* Explicitly described physical, verbal or sexual abuse during Adult	6, 12, 14, 15, 17, 19
*3. Family patterns of distress and/or dysfunction* Expressions of illnesses, behaviours, or conditions that were repeated among different family members; also includes expressed negative emotions about the actions and intentions of family members and other personal relationships	1, 3, 4, 5, 6, 8, 9, 10, 11, 13, 14, 15, 16, 17, 18, 19
**Perceptions**	
*4. Entitlement* Inferred lack of participant's sense of accountability for his or her actions or inactions, usually from statements that offer symptoms as excuses for not being able to fulfil societal roles	1, 3, 4, 6, 10, 14, 16, 17, 18
*5. Health anxiety* Expressed or inferred participant concern about serious undiagnosed disease. Neither normal tests nor doctors' benign assessments of their symptoms reassured patients who expressed health anxiety. Either they had personally experienced a medical error, or they knew someone who had	3, 5, 7, 12, 19
*6. Psychological explanations and insights* Expressed or inferred awareness of the relationship between personal psychological stress and physical symptoms	5, 7, 8, 11, 12, 15 - *"high" insight* 2 - *"neutral"* 1, 4, 6 - *"very low" insight* All others - *"low" or "moderate" insight*
**Behaviours**	
*7. Symptom focus* A pervasive emphasis on symptoms	1, 4, 6, 9, 10, 13, 14, 18, 16, 19
*8. Expressing dissatisfaction with healthcare* Expressed and inferred dissatisfaction with healthcare system or providers.	3, 7, 9, 11, 12, 13, 14, 16, 19
*9. Achievement* Expressions of higher education, supervisory role, professional status, entrepreneurship, and/or creative activities	2, 3, 5, 7, 8, 9, 10, 11, 12, 13, 14, 15, 16, 17, 18, 19
*10. Action* Expressed or inferred ability to cope effectively or change behaviour for the better	2, 3, 5, 7, 8, 9, 11, 12, 13, 15, 16, 19
*11. Altruism* Spontaneous descriptions of volunteer activity, significant care-taking or meaningful work	3, 5, 7, 8, 9, 11, 12, 15, 19

### Coping high utilisers (Participants 2, 8, 11, 15)

All patients in this group, except Participant 2, had current and/or past family dysfunction (see Table 3Themes 1, 2, 3) yet they all had achieved significant success in their lives and a degree of psychological insight. We found no evidence in their transcripts that any of these participants had previously been labelled with MUS by their providers. Three of the four participants in this group had moderate chart-rated MUS. Basic demographic characteristics are listed in Table 4. A typical member of the group, Participant 15, was a 59 year old female who mentioned in her opening statement that she had been raised in a dysfunctional family. Her father drank and fought a lot with her mother, and she felt hassled as a middle child. She was molested by her father's best friend when she was seven years old and subsequently endured 2 abusive husbands. Yet, like all others in this group, she left the impression that any resulting internal conflicts had been resolved - *"I have now dealt with it (sobbing), I have; I have been in therapy for it"*

**Table 4:** Three patient consultation groups with demographic and clinical characteristics

**Characteristic**	**Coping high utilisers (n = 4)**	**Classic high utilisers (n = 9)**	**Worried high utilisers (n = 6)**
Identification numbers	2, 8 11, 15	1, 4, 6, 9, 10, 13, 14, 17, 18	3, 5, 7, 12, 16, 19
Mean age (standard deviation)	54.5 (9.8)	47.0 (10.9)	53.2 (6.9)
Female gender N (% of patient group)	3 (75)	8 (89)	5 (83)
≤ 12 years education	3	2	0
14 years education	1	4	1
≥ 16 years education	0	3	5
Number of chart-rated severe MUS (%)	1 (11)	5 (56)	3 (33)
Number of chart-rated moderate MUS (%)	3 (30)	4 (40)	3 (30)
Mean number of visits/year for 2 years (standard deviation)	11.0 (2.7)	11.4 (3.1)	16.0 (9.6)

Mean proportion of visits MUS (standard deviation)	0.68 (0.24)	0.66(0.22)	0.74 (0.02)

Three (Participants 8, 11, 15) of the four coping high utilisers were both emotionally expressive and psychologically insightful (see Table 3). For example, Participant 15 sobbed appropriately as she shared painful memories, and engaged pleasantly in lighter parts of the conversation. She talked about learning to support her adult children without assuming blame for their bad choices. Similarly, Participant 8, a 54-year old female, believed strongly that effective coping required the right *"attitude." *She found it remarkable that *"no one [in her family] ever talked." *She, on the other had, *"talked about everything." *Participant 11, a 47-year old male learned from his brother that, *"most of the problems [he had were] up here *(pointed to head), *and if some day [he could] control that, then [he] wouldn't have the problems... the aches and pains come with that." * Although she was felt to be neutral with respect to Theme 6 (psychological insight, see Table 3), Participant 2, the other coping utiliser, described herself as a happy and content person with excellent relationships. She was visibly happy, especially when she talked about her husband.

Coping high utilisers did not focus on their symptoms or appear to feel entitled (see Table 3). When they did talk about symptoms, their descriptions were concise. For example, in describing her back pain a recently retired factory worker (Participant 15) explained, *"I would hurt within two hours [of bending over and underneath cars at work]." *Moreover, coping high utilisers were resourceful (see Table 3, Themes 9 and 10) and/or altruistic (Theme 11). For example, in addition to quitting a lifelong habit of heavy drinking, Participant 15 was able to quit smoking and later, to adopt a diabetic diet. She enjoyed her work and planned to volunteer after her upcoming retirement. Unlike participants who sought excuses to miss work, she actually felt ambivalent about retiring. Similarly, Participant 8 wrote poetry and managed a local store while pursuing a professional degree. She described how she engaged in various recreational activities to help cope with illness; and she was altruistic (as were Participants 11 and 15) -*"I was there when [my stepfather] had his surgery... My oldest brother suffers from post-traumatic stress syndrome... I have been doing a lot to help him."*

Despite their ability to function, these patients had a mean of 11 visits per year for 2 years prior to enrolment (see Table 4). The majority of these visits (68%) were for chart-rated MUS. Although all subjects in the study had at least two years of high utilisation (most had three), it is possible that at the time of the interview, these patients had improved on the basis of treatment and/or other factors and were on the way to low utilisation. For example, Participant 15 had experienced lot of musculoskeletal symptoms during initial recruitment for the randomized controlled trial, but she noticed fewer symptoms after retiring from her factory job shortly before the interview. Some visits were the result of delay, or difficulty in diagnosis. For example, Participant 8 had multiple consultations, testing and referrals for excruciating chest pain. Eventually, she was told she had a leaking breast implant, although no definitive diagnosis was made before breast implants were surgically removed. Many of these visits were driven by the provider for the purposes of diagnosis, treatment, and/or monitoring. All participants had co-morbid medical or psychiatric disease that required periodic monitoring and need for medication. Visits that were previously scheduled for monitoring organic disease may have been used to assess or monitor a self-limiting acute illness. For example, Participant 15 who had visited the doctor the day before for shoulder pain said, *"He put me on some medication...for two weeks because I have to go back and see him in two weeks, because he is um, I have to go back every three months for my blood sugar." *Thus, a visit designated "primarily MUS" on the basis of documented consultation activity may have originally been scheduled by the physician for follow-up of organic disease.

Coping high utilisers did not appear to have significant health anxiety (see Table 3, theme - health anxiety); and some claimed to generally limit the number of times they sought consultations. For example Participant 8 noted that she was not *"the type of person who runs to the doctor every time I get a pain. It's really got to be excruciating, you know, where I think, ok, this has been going on for a couple of weeks." *However, they admitted to having unmet cognitive needs concerning diagnosis, treatment, and/or prognosis. For example, Participant 2 denied that she was worried about misdiagnosis but said, *"I guess my main concern is, yes, an explanation. Once I have an explanation, then if there's a treatment process, then people know where to go with it. As long as it is unexplained, then there's a question in my mind and how do you treat something that you don't know what it is?" *Similarly, Participant 8 was not impressed with the explanation given for an incidental finding after multiple chest x-rays: *"I don't know how many x-rays I had that year... I told the technician, "I ought to glow in the dark... They told me it was inactive disease. How can I have an inactive disease when I never had an active disease?" *Her scepticism was evident in another section of her narrative: *"That is your standard answer, you know. "Well, we really don't know, understand fully these things." And I am like, you know, okay!*" Despite their unmet needs, all coping high utilisers reported having good relationships with their current providers. Only Participant 11 expressed dissatisfaction with his healthcare provider(s) (see Table 3, Theme 8) by stating that he had considered leaving the practice because some providers lectured him about his use of pain medications.

### Classic high utilisers (Participants 1, 4, 6, 9, 10, 13, 14, 17, 18)

Like coping high utilisers, these participants had current and/or past family dysfunction (see Table 3, Themes 1, 2, 3). However, these classic high utilisers also perseverated on their vague symptoms (Theme 7), demonstrated little psychological insight (Theme 6) and/or expressed strong entitlement (Theme 4) that they should be excused from normal social obligations. Six of the nine participants had previously been labelled with an MUS diagnosis (five with fibromyalgia and one with irritable bowel syndrome). One had been told she had an "autoimmune disease" from breast implants and two had not received any MUS label. Of the two who had not been previously labelled (Participants 13 and 17), one had chronic neck and back pain which the patient attributed to a motor vehicle accident in 1996. Both of the unlabelled patients had moderate chart-rated MUS, suggesting that most of their MUS for the recruitment period were minor self limited illnesses. Five of the nine classic high utilisers had severe chart-rated MUS and four had moderate MUS. Seven of them had completed at least 14 years of formal education. Other demographic characteristics are listed in Table 4.

Unlike coping high utilisers, these patients typically did not appear to have recovered from the traumatic experiences they talked about. One middle aged female part-time insurance agent (Participant 4) said she was inexpressive emotionally because "*I think I shut my feelings off quite a while ago. My dad died when I was 10..." *Participant 1, a 44-year-old single mother described how she and her siblings tried to cope with constant parental discord: *"I can remember being a little kid and they would come home late at night screaming and arguing and throwing things. We'd wake up and that was kind of scary to go through that..." *Asked how she and her siblings responded, she simply said, *"We would try to go back to sleep." *As an adult, she continued to use avoidance, *"I come home from work and everything's a mess and I don't want to poke in the mess and figure out what... I just escape." *Participant 6 admitted there were a lot of unresolved issues in her family, *"there are a lot of things that I probably haven't even told my family that I went through, my brothers and my sisters, but I went through a lot." *Participant 18, whose affect had been flat through out the interview, was visibly upset when she said: *"mom and dad would argue sometimes, and I absolutely hated to see it and I hate dissension... Dad will never argue until mother just, you know, pushed him so far, and dad was soft spoken; mother was argumentative, she, and I think she is... had a mental problem." *Participant 9, a 41-year old female administrator who was adopted when she was two years old, was still disappointed by parental favouritism, "*They can never give me anything materialistic that would equal what they sacrificed for [sister]"*; and neglect, *"They had an opportunity to take care of me... but they didn't, and I have a lot of struggles from that." *Participants 1, 4, 6, and 18 all described periods of loneliness and isolation, and at least one suggested that she went to her doctor because she didn't think it was fair to talk to anyone else about her problems.

Unlike the past trauma of coping high utilisers, some of the experiences (Themes 1, 2, 3) described by classic high utilisers were temporally linked to their physical symptoms. For example, Participant 14 who was raped and abused physically by her boyfriend when she was 16 years old subsequently developed chronic pelvic pain. Similarly, Participant 6 suffered physical abuse at the hands of two consecutive husbands and reported concurrent medically unexplained chronic musculoskeletal pain for over 12 years. Surprisingly, she seemed unable to imagine a possible association with abuse, focusing instead on a previous motor vehicle accident. When asked directly if she felt her husband's abuse might have contributed in any way to her symptoms, she said, "*I had no lingering pain from anything he did..." *This apparent lack of general psychological insight was typical of classic high utilisers (see Table 3, Theme 6), who wove and coupled descriptions of symptoms with descriptions of their jobs, housework, spouses, and/or children, with no evidence that they were aware of any possible associations.

Transcripts of all labelled classic high utilisers also revealed a pervasive emphasis on vague symptoms (see Table 3, Theme 7). The following transcript fragment from Participant 10 who had fibromyalgia for over 10 years illustrates how some patients overwhelmed their providers with their chaotic narratives: *"I go in and I say, 'I can't sleep, and it kind of comes and goes... ' One thing goes out of whack and I feel a lot of things so I come in a lot of times and ... I need to get them all in, I need to tell her everything that is going on... I think in the beginning I might have overwhelmed her." *The patients talked about how painful and debilitating their symptoms were, often in the context of their care-seeking and/or their normal social roles. They reported often that they could not or would not perform normal social obligations like housework or grocery shopping (Theme 4) because of pain or fatigue. Participant 6 summed up their collective attitude, "*I'm just gonna feel rotten today and not do very much. I am in pain; the more I do, the worse the pain gets, but I will just take the day off." *They talked about how they would pay later with more symptoms if they relented and engaged in normal recreational activities or chores. A few patients expressed regret about this, but more often, they expected to be excused and/or to be relieved by reluctant family members (see Table 3; Theme 4). Thus, compared with coping high utilisers, classic high utilisers appeared to be much more disabled by their symptoms. Indeed Participants 1, 4, and 6 (who had the highest ratings on symptoms focus, entitlement, and lowest on psychological insight) did not demonstrate any achievement, action, or altruism (see Table 3; Themes 9, 10, 11). Instead of finding meaning in their work, they talked about getting time off, or quitting their jobs because of their debilitating symptoms.

As with coping high utilisers, utilisation among these patients was driven primarily by multiple testing, referrals, and empirical treatments. For those who were eventually given a label, diagnosis was often delayed by at least two or three years. Typically, they saw multiple doctors who tried different things, ordered many tests, referred them to other doctors, until eventually someone *"found trigger points" *or simply labelled them as having fibromyalgia. Unfortunately, eventual diagnosis was not coupled with any perceived change in management or strategy. According to Participant 18: *"No [the doctor's management didn't change], I never really did anything [different] except try to take really good care of myself, rest, eat properly, which I kind of always have anyway." *Participant 14 suggested her doctor made the diagnosis for a purely pragmatic reason, *" [he said], 'I know you are...that you have pain and I know that you have physical representations of that pain. I feel your back, I feel everything out of whack, I know...' he goes, 'Am I absolutely positively sure that it's fibromyalgia?' 'No, I am not.' He goes, 'but that is a diagnosis; it does get you through the system, and you know, then we don't have to deal with all the other crap'..." *Not surprisingly, she was not happy with the diagnosis: *"I actually had hoped at one point that I had something different, because then it would have been fixable. I was a little bit upset, actually, when I saw the diagnosis for the fibromyalgia because it is a very symptom-based thing and you know, they don't know what causes it, they don't know this, they don't know that. And I was sort of like 'why are you giving me that diagnosis?"'*

All others were relieved, at least initially, to finally have a diagnosis. As Participant 18 said to herself, *"Hah there is a reason. I am not losing my mind!" *Yet, these labelled patients continued to seek care, often for help with managing their symptoms. For example, Participant 6 said, "*sometimes I go in, because I hurt so badly. I had real bad headaches and neck aches and you know, he said, well, you know, you got fibromyalgia. What am I gonna say? Haha... You know, well, take it out. Haha... You know, can't you cure me of this mess? And I won't take a lot of pills" *Participant 18 also desired something other than medications, *"I don't want more drugs but I want pain management." *Some patients wanted suggestions for self-management and/or wondered privately whether they would benefit from procedures or surgery. Participant 1 also wished (as did several others) that she had more time "*to sit down and tell a story more like this [interview]. Sit down and say this is what's happening to me." *She wanted more guidance: "*I feel like doctors come in the room and they talk to you and they give you medicine and they leave and the rest of it is up to you." *She explained how after one sequence of multiple testing, referrals and empiric treatments, she finally realized (without the help of her doctors) that she needed a different approach: *"I decided that if everything is normal [with her shoulder], then I need to take control or be more in charge, take on a new attitude. I have to drop this, "there's something wrong, I need surgery again," which is something that I felt through all the back and forth and all the tests. So, I decided to bury that, because obviously it wasn't true. And, I started to take myself off of the pain medication." *This showed that when present, psychological insight, though rare, was beneficial even in this group. Unfortunately, this patient's breakthrough did not curtail clinic visits for her other symptoms.

Participant 14, a law school graduate, was uniquely assertive among this group; and she clearly felt entitled to seek all the care she desired, "*my place of employment pays very good money for me to have this kind of health care. And I want my health care; I don't want to be told I don't need this." *None of the other classic high utilisers displayed this particular kind of entitlement.

### Worried high utilisers (Participants 3, 5, 7, 12, 16, 19)

Members of this group had characteristics of coping high utilisers (Participant 7), classic high utilisers (Participant 16), or both (Participants 3, 5, 12, 19). Similarly, half had moderate chart-based MUS and half had severe chart-based MUS (see Table 4). However, unlike members of either coping or classic high utilisers, all except Patient 16 had health anxiety (Theme 5); and all except Patient 5 complained about their healthcare (Theme 8). On average they had 16.0 (standard deviation = 9.6) visits with 75% MUS/visit. They all had at least 14 years of formal education (five had 16 or more); and only one (Participant 7) had previously been labelled with MUS (IBS).

Participant 12, the highest attendee of all 19 patients, was a highly accomplished 52 year old female with unlabelled MUS, who visited her primary care provider 35 times/year for *"a lot of ongoing little things;" *85% of these visits were for MUS. She knew of no co-morbid chronic medical or psychiatric disease. She talked about how unpleasant it was to watch her mother die from misdiagnosed metastatic cancer and reported other significant family history of cancer and cardiovascular disease. She was candid both about her anxiety, "*you look at your history and you think, 'oh, man, you're a walking disaster here, waiting to happen... When something happens, if I get a toe injury or whatever of course the first thing you think is "Oh my God I'm..." *and about how the anxiety caused her to demand excessive care: *"you think gee if they could miss it twice. That's a pretty scary thing and you know, in fact, a little while ago, I insisted that they take out a lump; I've had like five lumpectomies. And you know they said, 'it's nothing,' you know, 'you don't need to worried about it.' I went to see [surgeon], and he examined it and he did a needle biopsy, and said, 'I think it is fine.' And I said: 'I think' isn't good enough. I want it out of there." *Participant 7(who had 14 visits/year) also was frank about how anxiety drove her utilisation: *"I get too nervous, stressed, and then I feel all kind of things, you know...sometimes I wonder myself if my symptoms are real, you know. But I do feel them... I get the anxious, you know, and then everything that happens, for example, if I get anything that wasn't here, then I want to see the doctor because I am worried that it could be something bad. I mean that not that I am making up things because for example, if I have a swollen ankle, I'm not making it up, it's there. But um sometimes I think that I worry too much..." *Interestingly, neither of these patients remembered talking openly with their providers about their anxieties or the reasons for them. When asked directly whether she had discussed one of her fears with any of her doctors or nurses, Participant 7 said, *"No. They don't spend time with you; they don't; they are in a hurry, always. They are in a hurry. When they tell me that I have a doctor, I don't feel like I have a doctor..." *She went on to complain about how difficult it was to see her own doctor. All worried high utilisers expressed similar dissatisfaction (Theme 8), usually related to limited access to healthcare. Participant 9 wondered whether her providers had a financial conflict of interest, *"Do you get an extra little money in your pay check or something for finding disease; I mean, what's the scoop here, you know?" *Similarly, Participant 7 thought that the only reason she was expected to see a nurse practitioner for routine gynaecological care instead of a specialist was *"to save money, because that is the only thing, there is no other excuse, it is just to save money." *She also believed her providers were reluctant to order tests, because they were *"following some orientation [from insurance companies]." *When asked how she felt about this, she said, "*I think this is bad... I feel helpless when it comes to doctors because I don't know where to go to complain... Frustration is the biggest word in my life, just about everything." *And this patient, at least, was convinced that "frustration" was the root cause of her symptoms: *"As a result of all the frustration, I have high cholesterol level, I get too nervous, stressed, and then I feel all kind of things..."*

## Discussion

Our study focused on understanding MUS through the lived experiences and attitudes of high utilising primary care patients. Because there are no agreed-upon research criteria for primary care patients with MUS[34], it can be argued that the themes we have generated might relate to frequent attending in general, high utilisation of care in general, medically unexplained symptoms in general, or some other common characteristic of the sample that may be unrelated to either. However, these are not mutually exclusive groups; and while they may be useful for research purposes, the distinctions are likely to be of limited clinical value. Although MUS is common in the outpatient setting, it is not generally considered a problem until it leads to frequent health seeking and excessive utilisation. Our unique database and definition of MUS allowed us to investigate the perspectives of high utilising patients with unlabelled or unrecognized MUS and to integrate those perspectives with our growing knowledge of primary care patients with MUS.

We found that current or past family dysfunction was a common feature of all three subsets in this sample. This is consistent with previous quantitative studies that have documented an association between a history of abuse and MUS [35-37]. It is not clear how such emotional trauma leads to MUS or high utilisation. In our study, patients who had not addressed their past trauma were much more disabled by their symptoms. Conversely, those with increased psychological insights and some resolution of emotional trauma were more successful in life and focused less on symptoms. Yet, they continued to engage in excessive utilisation.

We identified three distinct patterns of perceptions and behaviours among these high utilising patients with MUS. Coping high utilisers were patients with current or past history of abuse and/or other family dysfunction who had achieved success in their lives and a degree of psychological insight. They neither focused on their symptoms nor displayed significant health anxiety. They were not afraid of having undiagnosed terminal disease, but they wanted explanations for their symptoms. As is often the case with patients in such "diagnostic limbo,"[20] high utilisation was driven primarily by futile quests for organic diagnosis and ineffective empirical therapies. Previous studies have suggested that such excessive testing and ill-advised empirical treatments lead to iatrogenic complications and increased costs with little relief for patients with MUS [18,38,39]. Effective treatment for MUS in primary care exists [29,40,41], but before patients can benefit from such treatments, MUS must first be diagnosed and effectively explained to patients. General practitioners who are motivated and trained to treat patients with MUS have identified as an important barrier to treatment, not being able to say definitively that patients have MUS [12]. Clearly, there is a need to recognize MUS at an earlier stage in order to begin treatment and reduce utilisation [18,34]. This requires management strategies that acknowledge and incorporate the inherent uncertainty of MUS diagnosis. Both clinicians and their patients will be better served by understanding that MUS is a real (and common) condition that should be considered right from the initial consultation as part of the differential diagnoses for most symptoms [42]. Providers must also learn to recognize and address cues given by patients about the kind of explanations [43] they seek.

A second group of patients with similar familial dysfunction were much more troubled by their symptoms and displayed much less psychological insight. Most of these patients had been told they had MUS and were satisfied with their generally limited understanding of what that meant. As in other studies [44], their initial relief gave way to disappointment as they discovered the limitations in the treatment options and understanding for their newly discovered diagnoses. Nevertheless, these chronic MUS patients continued to consult their doctors for medical and social support. For many patients with MUS, legitimization of symptoms by friends, family members, and health professionals was more important than having a diagnosis [45,46]. In reviewing the interview transcripts, we saw many missed opportunities for negotiation of the sick role with patients, families, and doctors.

When present, heightened health anxiety had an incremental effect on the utilisation of both coping and classic high utilisers, paralleling the results of an earlier qualitative study of somatising patients [47]. These worried high utilisers became angry and complained when they perceived resistance to their expectations and demands. To effectively treat these patients, their doctors needed to recognize and understand the source of their anxiety and to use this as a focus to help to rebuild their trust. Primary care providers can be taught to use patient-centred skills to recognize which patients have health anxiety, to express empathy, and to guide patients who demand excessive care in more effective medical decision-making. This approach has been effective in treating similar patients with MUS [29].

If corroborated, the distinct patterns of consultation and needs we identified will have important implications for primary care management of patients with MUS. Although current protocols call for individualization of treatment, their core principles assume a much more homogeneous group than our study would suggest. For example, Smith et al advocate education, commitment, goals, and negotiation at every visit for all high utilizing MUS patients [28]. Similarly, "making the link explanations" are universally recommended in the reattribution model [48]. However, our study suggests a more selective use based on individualized, patient-centred interactions because patients may benefit variably from rote use of all approaches. We suggest that primary care practitioners determine from patients' perceptions and behaviours the most propitious groups for different intervention strategies. This could significantly improve both the effectiveness and efficiency of consultations with patients with MUS.

We must acknowledge important limitations. Our findings, like other qualitative studies, may not be applicable to other primary care patients with MUS, such as those with very severe disease who could not or would not be willing to take part in a randomised controlled trial. Secondly findings from this qualitative study are subject to the biases of the investigators. However, our sampling strategy and method of iterative consensus building and emergent adjustments to our design allowed us to explore the broadest possible range of experiences and behaviours related to MUS. We also recognize that relying on patient report, rather than direct observation may limit the content validity of some of our themes, especially those purported to describe experiences and behaviours. Nonetheless, the information obtained from our interviews more closely approximates the information available to primary care clinicians, and therefore, may be more useful clinically. Finally, we recognize that the typology of the 3 groups was not perfect. For example Participant 11, was not classified as a worried high utiliser, even though she expressed dissatisfaction (theme 8). This is not surprising considering that the groups emerged qualitatively. Indeed the consistency of themes in these qualitatively determined groups is impressive and argues for their validity. Nevertheless, it is possible that the most we can conclude from our data is that patients with high utilising MUS present with different combinations of several themes. However, describing the themes on the basis of the three groups allowed hypotheses to emerge from the data by provoking questions like, 1) why did patients with psychological insights and coping skills continue to seek care; and 2) why would patients who were so dissatisfied with their healthcare continue to consult their providers?

One of the singular values of qualitative research is hypothesis generation; this study has generated several hypotheses that will benefit from quantitative assessment:

1) Patients with chronic MUS with severe disability, and low psychological insight will benefit from treatment that emphasizes legitimization, support and guidance with self-management and role-negotiation rather than reattribution or symptom explanation.

2) MUS patients who endorse psychological explanations and insights may have better coping mechanisms, and may be the most propitious group for treatment that emphasizes plausible explanations that are acceptable to the patient.

3) Excessive complaints about access to healthcare is a marker for unrecognized or unexpressed worry in some high utilising primary care patients with MUS. Training clinicians and patients to recognize and address the anxiety and its source will reduce cost in this subset of patients.

## Conclusion

This qualitative descriptive study identified three distinct consultation patterns among high utilising primary care patients with MUS. Coping high utilisers with psychological insight demonstrated less disability, but continued to have high utilisation primarily because of ineffective biomedical approaches. Classic high utilisers without psychological insight displayed more disability and continued to seek care for relief of symptoms and for support. Health anxiety appeared to have an incremental effect on high utilisation regardless of patients' degree of insight or ability to cope.

## Competing interests

The authors declare that they have no competing interests.

## Authors' contributions

FCD conceived of and coordinated the study, participated in its design, interviewed all subjects, performed qualitative analyses and interpretations, drafted the manuscript, responded to reviewer comments, and critically revised the manuscript. JSL and RCS participated in the design of the study, qualitative analyses and interpretation of data, and critically revised the manuscript. RMF participated in the design of the study and interpretation of data and critically revised the manuscript for important intellectual content. All authors read and approved the final manuscript.

## Pre-publication history

The pre-publication history for this paper can be accessed here:



## References

[B1] Lipowski ZJ (1988). Somatization: the concept and its clinical application. Am J Psychiatry.

[B2] Peters S, Stanley I, Rose M, Salmon P (1998). Patients with medically unexplained symptoms: sources of patients' authority and implications for demands on medical care. Soc Sci Med.

[B3] Travers MK, Lawler J (2008). Self within a climate of contention: Experiences of chronic fatigue syndrome. Soc Sci Med.

[B4] Werner A, Isaksen LW, Malterud K (2004). 'I am not the kind of woman who complains of everything': illness stories on self and shame in women with chronic pain. Soc Sci Med.

[B5] Johansson EE, Hamberg K, Lindgren G, Westman G (1996). "I've been crying my way"--qualitative analysis of a group of female patients' consultation experiences. Fam Pract.

[B6] Katon WJ, Walker EA (1998). Medically unexplained symptoms in primary care. J Clin Psychiatry.

[B7] Dirkzwager AJ, Verhaak PF (2007). Patients with persistent medically unexplained symptoms in general practice: characteristics and quality of care. BMC Fam Pract.

[B8] Kirmayer LJ, Robbins JM (1996). Patients who somatize in primary care: a longitudinal study of cognitive and social characteristics. Psychol Med.

[B9] Hahn SR (2001). Physical symptoms and physician-experienced difficulty in the physician-patient relationship. Ann Intern Med.

[B10] Hahn SR, Kroenke K, Spitzer RL, Brody D, Williams JB, Linzer M, deGruy FV (1996). The difficult patient: prevalence, psychopathology, and functional impairment. J Gen Intern Med.

[B11] Wetterneck TB, Linzer M, McMurray JE, Douglas J, Schwartz MD, Bigby J, Gerrity MS, Pathman DE, Karlson D, Rhodes E (2002). Worklife and satisfaction of general internists. Arch Intern Med.

[B12] Dowrick C, Gask L, Hughes JG, Charles-Jones H, Hogg JA, Peters S, Salmon P, Rogers AR, Morriss RK (2008). General practitioners' views on reattribution for patients with medically unexplained symptoms: a questionnaire and qualitative study. BMC Fam Pract.

[B13] Nordin TA, Hartz AJ, Noyes R, Anderson MC, Rosenbaum ME, James PA, Ely JW, Agarwal N, Levy BT (2006). Empirically identified goals for the management of unexplained symptoms. Fam Med.

[B14] Salmon P, Ring A, Dowrick CF, Humphris GM (2005). What do general practice patients want when they present medically unexplained symptoms, and why do their doctors feel pressurized?. J Psychosom Res.

[B15] Ring A, Dowrick CF, Humphris GM, Davies J, Salmon P (2005). The somatising effect of clinical consultation: what patients and doctors say and do not say when patients present medically unexplained physical symptoms. Soc Sci Med.

[B16] Peters S, Rogers A, Salmon P, Gask L, Dowrick C, Towey M, Clifford R, Morriss R (2009). What do patients choose to tell their doctors? Qualitative analysis of potential barriers to reattributing medically unexplained symptoms. J Gen Intern Med.

[B17] Barsky AJ, Borus JF (1999). Functional somatic syndromes. Ann Intern Med.

[B18] Smith RC, Dwamena FC (2007). Classification and diagnosis of patients with medically unexplained symptoms. J Gen Intern Med.

[B19] Salmon P, Ring A, Humphris GM, Davies JC, Dowrick CF (2009). Primary care consultations about medically unexplained symptoms: how do patients indicate what they want?. J Gen Intern Med.

[B20] Nettleton S, Watt I, O'Malley L, Duffey P (2005). Understanding the narratives of people who live with medically unexplained illness. Patient Educ Couns.

[B21] Smith RC, Gardiner JC, Lyles JS, Johnson M, Rost KM, Luo Z, Goddeeris J, Lein C, Given CW, Given B (2002). Minor acute illness: a preliminary research report on the "worried well". J Fam Pract.

[B22] Smith RC, Gardiner JC, Lyles JS, Sirbu C, Dwamena FC, Hodges A, Collins C, Lein C, Given CW, Given B, Godderis J (2005). Exploration of DSM-IV criteria in primary care patients with medically unexplained symptoms. Psychosom Med.

[B23] Glenton C (2003). Chronic back pain sufferers--striving for the sick role. Soc Sci Med.

[B24] Smith RC, Korban E, Kanj M, Haddad R, Lyles JS, Lein C, Gardiner JC, Hodges A, Dwamena FC, Coffey J, Collins C (2004). A method for rating charts to identify and classify patients with medically unexplained symptoms. Psychother Psychosom.

[B25] Charmaz K (1990). 'Discovering' chronic illness: using grounded theory. Soc Sci Med.

[B26] Huberman AM, Miles MB (1983). Drawing Valid Meaning from Qualitative Data: Some Techniques of Data Reduction and Display. Quality and Quantity.

[B27] Strauss A, Corbin J (1990). Basics of qualitative research Grounded theory procedures and techniques.

[B28] Smith RC, Lein C, Collins C, Lyles JS, Given B, Dwamena FC, Coffey J, Hodges A, Gardiner JC, Goddeeris J, Given CW (2003). Treating patients with medically unexplained symptoms in primary care. J Gen Intern Med.

[B29] Smith RC, Lyles JS, Gardiner JC, Sirbu C, Hodges A, Collins C, Dwamena FC, Lein C, William Given C, Given B, Goddeeris J (2006). Primary care clinicians treat patients with medically unexplained symptoms: a randomized controlled trial. J Gen Intern Med.

[B30] Kleinman A (1988). The Illness narratives - suffering, healing and the human condition.

[B31] Seville JL, Robinson AB, RJ G, Weisberg JN (2000). Locus of control in the patient with chronic pain. Personality characteristics of patients with pain.

[B32] Fink P (1993). Admission patterns of persistent somatization patients. Gen Hosp Psychiatry.

[B33] Crabtree BF, Miller WL (1991). A qualitative approach to primary care research: the long interview. Fam Med.

[B34] Olde Hartman T, Hassink-Franke L, Dowrick C, Fortes S, Lam C, Horst H van der, Lucassen P, van Weel-Baumgarten E (2008). Medically unexplained symptoms in family medicine: defining a research agenda. Proceedings from WONCA 2007. Fam Pract.

[B35] Drossman DA, Leserman J, Nachman G, Li ZM, Gluck H, Toomey TC, Mitchell CM (1990). Sexual and physical abuse in women with functional or organic gastrointestinal disorders. Ann Intern Med.

[B36] Lipowski ZJ (1987). Somatization: the experience and communication of psychological distress as somatic symptoms. Psychother Psychosom.

[B37] Lown EA, Vega WA (2001). Intimate partner violence and health: self-assessed health, chronic health, and somatic symptoms among Mexican American women. Psychosom Med.

[B38] Fink P (1992). Surgery and medical treatment in persistent somatizing patients. J Psychosom Res.

[B39] Hoffman RM, Wheeler KJ, Deyo RA (1993). Surgery for herniated lumbar discs: a literature synthesis. J Gen Intern Med.

[B40] Morriss R, Gask L, Ronalds C, Downes-Grainger E, Thompson H, Leese B, Goldberg D (1998). Cost-effectiveness of a new treatment for somatized mental disorder taught to GPs. Fam Pract.

[B41] Morriss RK, Gask L (2002). Treatment of patients with somatized mental disorder: effects of reattribution training on outcomes under the direct control of the family doctor. Psychosomatics.

[B42] Sharpe M, Carson A (2001). "Unexplained" somatic symptoms, functional syndromes, and somatization: do we need a paradigm shift?. Ann Intern Med.

[B43] Salmon P, Wissow L, Carroll J, Ring A, Humphris GM, Davies JC, Dowrick CF (2008). Doctors' attachment style and their inclination to propose somatic interventions for medically unexplained symptoms. Gen Hosp Psychiatry.

[B44] Undeland M, Malterud K (2007). The fibromyalgia diagnosis: hardly helpful for the patients? A qualitative focus group study. Scand J Prim Health Care.

[B45] Nettleton S (2006). 'I just want permission to be ill': towards a sociology of medically unexplained symptoms. Soc Sci Med.

[B46] Salmon P (2000). Patients who present physical symptoms in the absence of physical pathology: a challenge to existing models of doctor-patient interaction. Patient Educ Couns.

[B47] Morse DS, Suchman AL, Frankel RM (1997). The meaning of symptoms in 10 women with somatization disorder and a history of childhood abuse. Arch Fam Med.

[B48] Morriss R, Dowrick C, Salmon P, Peters S, Rogers A, Dunn G, Lewis B, Charles-Jones H, Hogg J, Clifforda R, Iredale W, Towey M, Gask L (2006). Turning theory into practice: rationale, feasibility and external validity of an exploratory randomized controlled trial of training family practitioners in reattribution to manage patients with medically unexplained symptoms (the MUST). Gen Hosp Psychiatry.

